# PI4K-beta and MKNK1 are regulators of hepatitis C virus IRES-dependent translation

**DOI:** 10.1038/srep13344

**Published:** 2015-09-01

**Authors:** Joachim Lupberger, Claudia Casanova, Benoit Fischer, Amelie Weiss, Isabel Fofana, Nelly Fontaine, Toshinobu Fujiwara, Mickael Renaud, Arnaud Kopp, Catherine Schuster, Laurent Brino, Thomas F. Baumert, Christian Thoma

**Affiliations:** 1 Inserm U1110, Institut de Recherche sur les Maladies Virales et Hépatiques Strasbourg, France; 2 Université de Strasbourg, France; 3 Department of Medicine II, University of Freiburg, Freiburg, Germany; 4 High Throughput Screening platform, IGBMC, UMR7104 CNRS UdS, Inserm, U964, Illkirch, France; 5 Laboratory of Hygienic Chemistry, Graduate School of Pharmaceutical Sciences, Nagoya City University, Nagoya, Japan; 6 Institut Hospitalo-Universitaire, Pôle Hépato-digestif, Nouvel Hôpital Civil, Strasbourg, France

## Abstract

Cellular translation is down-regulated by host antiviral responses. *Picornaviridae* and *Flaviviridae* including hepatitis C virus (HCV) evade this process using internal ribosomal entry sequences (IRESs). Although HCV IRES translation is a prerequisite for HCV replication, only few host factors critical for IRES activity are known and the global regulator network remains largely unknown. Since signal transduction is an import regulator of viral infections and the host antiviral response we combined a functional RNAi screen targeting the human signaling network with a HCV IRES-specific reporter mRNA assay. We demonstrate that the HCV host cell cofactors PI4K and MKNK1 are positive regulators of HCV IRES translation representing a novel pathway with a functional relevance for the HCV life cycle and IRES-mediated translation of viral RNA.

Hepatitis C virus (HCV) is a positive stranded RNA virus replicating in intracellular phospholipid-enriched membrane domains. Several unbiased RNAi screens identified a panel of host factors required for HCV entry, replication and assembly^1^,^2^,^3^,^4^ but none of these previous approaches discriminates effects on mRNA translation. Host protein translation is initiated with the recruitment of the 40S ribosomal subunit to mRNA. This process mostly involves the recognition of a 5′ m7GpppN cap structure by eIF4E of the cap binding complex eIF4F^5^. Most eukaryotic mRNAs also contain a 3′ poly(A) tail, which is acting synergistically with the cap structure to enhance translation^6^,^7^,^8^. Initiation of cap-dependent translation is susceptible to regulation via eIF4F by eIF4E inhibitory proteins by phosphoinositide 3-kinase (PI3K)/Akt and mammalian target of rapamycin (mTOR) signaling pathways^9^. Furthermore, MAP kinase pathways modulate cap-dependent translation by phosphorylation of ribosomal protein S6^10^ and by eIF4E phosphorylation via MAP kinase interacting serine/threonine kinase 1 (MKNK1)^11^,^12^. Furthermore, cap translation is inhibited by heatshock proteins^13^ and by protein kinase R (PKR) and PKR-like endoplasmic reticulum kinase (PERK), which are activated by double stranded viral RNA intermediates and ER-stress, respectively^14^. PKR and PERK are thus triggered by a cell in despair trying to prevent viral RNA replication and to activate repair mechanisms that rely on an alternative translation initiation mechanism mediated by internal ribosomal entry sequences (IRESs). IRES translation is thus of particular physiological importance when cap-dependent translation is compromised^15^,^16^,^17^,^18^, but which is also used by some positive strand RNA viruses including HCV^5^,^19^,^20^ promoting viral protein synthesis^21^. It has been demonstrated that miR-122 stimulates HCV IRES translation^20^,^22^ and that RACK1 controls the IRES-mediated translation of viruses including HCV^23^ but additional host factors which are critical for HCV IRES activity remain largely to be determined. Since cellular signaling events regulate key aspects cap-dependent translation^9^, miRNA expression^24^ and the HCV life cycle^2^,^25^ we studied the role of host kinases and protein phosphatases in IRES-dependent translation.

## Results

**Figure 1:**
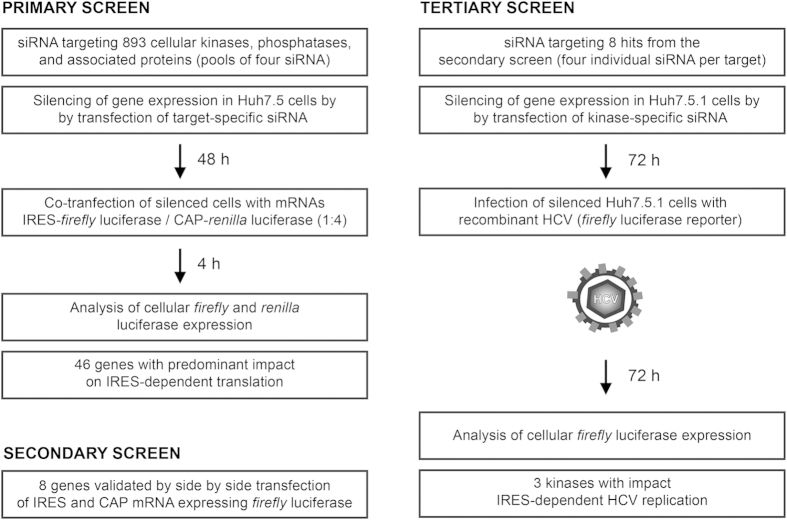
High-throughput RNAi screen identifying human kinases and protein phosphatases withpredominant impact on IRES-dependent translation and HCV infection.

**Figure 2:**
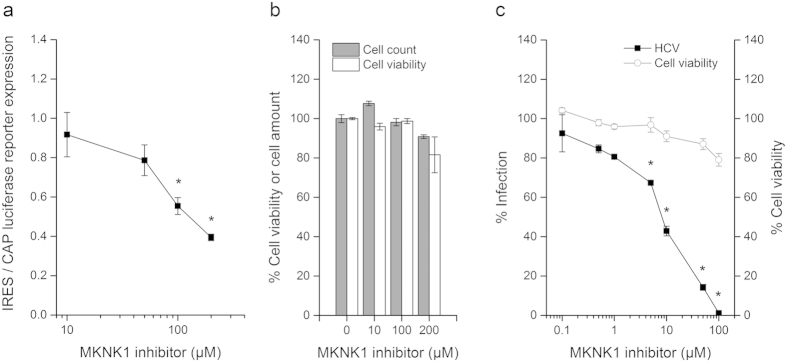
Kinase activity of MKNK1 promotes IRES-dependent translation and HCV infection. (**a**) A specific inhibitor of MKNK1 kinase activity preferentially impairs IRES-dependent over CAP-dependent luciferase reporter translation. Huh7.5 cells incubated for one hour with increasing doses of MKNK1 inhibitor. Treated cells were co-transfected with reporter mRNAs (IRES-*firefly* and cap-*renilla*) for four hours prior measuring of the *firefly* and *renilla* luciferase activity as described in the methods section. Data are expressed as means of the ratio of firefly/renilla luciferase activity +/− SEM. *p < 0.01 (Student’s t-test, n = 15 of three independent experiments). (**b**) MKNK1 inhibitor has only a minor impact on cell viability of Huh7.5 cells. After 5 h incubation with increasing concentrations of MKNK1 inhibitor the cell number was assessed by counting and the cell viability was assessed using Presto Blue. Data are expressed as means +/− SEM (n = 3 of one representative experiment). (**c**) MKNK1 inhibitor significantly and dose-dependently inhibits HCV infection at absent cell toxicity. Huh7.5.1 cells were pre-treated for one hour with increasing concentrations of MKNK1 inhibitor prior infection with cell culture-derived HCV (strain Luc-Jc1). Infected cells were maintained in the presence of the respective MKNK1 inhibitor concentration prior cell lysis and the measurement of the luciferase activity at day three. Data are expressed as means +/− SEM. *p < 0.01 (Student’s t-test, n = 3 of one representative experiment). All inhibitor dilutions and controls in this figure were prepared in a constant background of 1% DMSO.

To analyze the impact of gene silencing on IRES- and cap-dependent translation, respectively, we co-transfected reporter mRNAs (100 ng/0.3 cm^2^) in gene silenced hepatoma cells 48 h post siRNA transfection as described previously^26^,^27^ (Fig. 1): *Renilla* luciferase mRNA initiated by a m7G cap structure and *firefly* luciferase mRNA containing a non-physiological adenosine cap structure (‘A-cap’) and the HCV IRES element. The A-cap maintains stability of the mRNA, but is not recognized by the cap binding complex. Luciferase expression was assessed by a Mithras LB 940 (Berthold Technologies) using Dual-Luciferase Reporter Assay or Bright-Glo (Promega). Toxicity of gene silencing was assessed using MTT (Sigma) and Presto Blue (Sigma) for the tertiary screen. In the primary screen targeting 893 genes we identified 46 candidates that predominant impact HCV IRES-dependent over cap-dependent translation (Supplementary table S1). In a secondary validation screen using side-by-side transfection of *firefly* reporter mRNAs of cap and HCV IRES (Fig. 1) we validated 11 hits of the primary screen (Supplementary table S2) and thus confirmed that these genes predominantly affect HCV IRES- rather than cap-dependent translation. As HCV IRES translation is a key step in the viral life cycle we assessed whether the identified genes confirm as positive regulators of HCV infection. We validated the results from the two foregoing screens (performed with siRNA pools) in a tertiary screen by at least two of four individual siRNAs per target (Fig. 1) to minimize off-target effects and validated mRNA knockdown specificity of the final hits by qPCR (Supplementary figure S1). As a result we confirmed that silencing of 3 genes from the secondary screening have a reproducible and significant impact on HCV infection: phosphatidylinositol 4-kinase catalytical subunit beta (PIK4CB), MAP kinase interacting serine/threonine kinase 1 (MKNK1), and tumor protein D52-like 3 (NYD-SP25) (Supplementary table S3). We were not able to validate a specific silencing of NYD-SP25 mRNA expression and therefore cannot rule out that off-target effects being responsible for the impact in IRES-dependent translation. Using a specific inhibitor of MKNK1^28^ we demonstrate a significant (p < 0.01, t-test) and preferential inhibition of IRES-dependent translation over cap-dependent translation of luciferase reporter genes (Fig. 2a) at absent cell toxicity (Fig. 2b). Long-term treatment with the MKNK1 inhibitor over three days significantly (p < 0.01, t-test) block HCV infection (Fig. 2c) demonstrating that the molecular mechanism of action of MKNK1 involves its kinase activity.

## Discussion

Understanding the IRES-mediated control of HCV protein synthesis is important for the understanding of HCV infection. Although much progress has been made in understanding HCV entry and replication translational control by the HCV IRES and the involved host factors remain largely unknown. For genes identified as HCV cofactors it was mostly not conclusive whether they affect HCV translation or -replication or both steps in the viral life cycle. An established unbiased multistep screening approach identified five genes modulating HCV IRES-dependent translation. Among the genes was MKNK1, a regulator of cap translation and recently described HCV entry factor^29^. MKNK1 regulates EIF4E affinity to cap mRNA in fibroblasts and is required for Herpes simplex virus (HSV-1) replication. For the first time we demonstrate that MKNK1 predominantly promotes IRES-dependent translation in hepatocytes (Supplementary table S1) that likely contributes to decreased core protein expression levels observed upon MKNK1 inhibition^29^. A specific MKNK1 inhibitor predominantly impairs IRES over cap-dependent translation demonstrating that the molecular mechanism of action of MKNK1 involves its kinase activity. Indeed, MKNK1 inhibitor specifically and dose-dependently impairs HCV infection highlighting its potential as antiviral target for IRES-dependent viruses. No phosphatase was identified suggesting that HCV IRES translation is mainly dependent on protein phosphorylation. Interestingly, the screen identified a phosphatidylinositol 4-kinase (PI4K), another key member of the phospholipid metabolism. PI4K generate membranes enriched in phosphatidylinositide 4-phosphate lipids, which serve as replication platforms for RNA viruses from the *Picornaviridae* and the *Flaviviridae*^30^. Strikingly, most members of these virus families replicate in such membranous platforms (poliovirus, coxsackievirus, Aichi virus, enterovirus, HCV)^30^,^31^ also rely on IRES-dependent translation. PI4K alpha and beta isoforms have been implicated in entry, replication and packaging of HCV^1^,^2^,^3^,^4^,^32^. Thus our data suggest an additional and previously unrecognized role of PI4K-beta for HCV IRES-mediated translation of the viral polyprotein and potentially also for other viruses that employ an IRES mechanism. Collectively, our data identify a novel pathway of HCV-host interactions with functional relevance for the HCV life cycle and IRES-mediated translation of viral RNA.

## Methods

### Transcription and transfection and RT-PCR

All transfections were performed at the High Throughput Screening platform of the Institut de Génétique et de Biologie Moléculaire et Cellulaire (IGBMC) in Illkirch, France. Gene silencing by RNAi for all screens was performed on 5000 cells/3.5 pmol siRNA/0.3 cm^2^ in 96 well plates by reverse transfection using Interferin (Polyplus Transfection) as described previously^2^,^25^. Non-targeting scrambled siRNA (Qiagen) and RISC-free siGlo RNA (Dharmacon) were transfected as mock controls. 100 ng/0.3 cm^2^ reporter mRNA was transfected 48 h post siRNA transfection using TransMessager reagent (Qiagen) as described previously^26^. *In vitro* transcription of mRNAs were described previously^27^. Reporter mRNAs were co-transfected in a ratio (IRES-*firefly*: cap-*renilla*) of 1:4 in the primary screen. In the secondary screen reporter mRNAs (IRES-*firefly*, cap-*firefly*) were transfected side-by-side. Luciferase expression was assessed by a Mithras LB 940 (Berthold Technologies) using Dual-Luciferase Reporter Assay System System or Bright-Glo (Promega). Toxicity was assessed using MTT assay (Sigma) and Presto Blue assay (Sigma) for the tertiary screen and the MKNK1 inhibitor experiments. RNA was extracted using RNeasy kit (Qiagen) and cDNA were generated using Maxima Reverse Transcriptase (Life Technologies). qPCR was performed using RT^2^ SYBR Green qPCR Mastermix (Qiagen) and a C1000 Touch Thermo Cycler (Bio-Rad).

### Cells culture, virus, DNA, siRNA and small molecules

Cell growth conditions of hepatoma cell lines Huh7.5 and Huh7.5.1 were described^33^,^34^. Infection with HCVcc (strain LUC-Jc1) was described^2^. siRNAs for the primary and secondary screen comprised the Human Kinase RNAi Set V2.0 (pool of four siRNAs) targeting 691 kinases and associated proteins and siRNAs targeting 203 human phosphatases from the Human Druggable Genome siRNA Set Version 4.0 (pools of four siRNAs) from Qiagen. Individual siRNA (four individual siRNAs per target) for the tertiary screen were obtained from Qiagen. The plasmids encoding *firefly* luciferase pT3FireflyLuc(pA), the *renilla* luciferase control plasmid pT3RenillaLuc(pA) and pHCV-IRES-luc has been described previously^27^,^35^,^36^. qPCR primers were obtained from Qiagen (RT^2^ qPCR assays). MKNK1 inhibitor was obtained from Calbiochem (Merck Millipore) and solved in DMSO.

### Screening hit selection

To evaluate the impact of gene silencing on IRES and cap-dependent translation, ratios of relative light units emitted by cells transfected with IRES *firefly* luciferase and capped *renilla* luciferase reporter mRNAs were formed and values normalized by the plate median of the relative light signals. Measurements were scored calculating z-scores using CellHTS2 software. Hits were assigned from the primary screen if z = <−1.29. Secondary screening results were normalized to % luciferase activity of control cells transfected with scrambled siRNAs. Hits were assigned if target silencing decreased IRES-dependent *firefly* luciferase activity and if the ratio of IRES- to cap-dependent *firefly* luciferase activities is <0.8. An impact of target-specific siRNA (four siRNA per target) on infection with HCVcc was identified in a tertiary screen using the method of strictly standardized mean differences (SSMD)^37^,^38^ using GUItars software^39^. A target gene with specific impact on HCVcc was considered a hit if at least two individual siRNAs impairs HCVcc infection with an SSMD < =−1.645 (“fairly strong” inhibition) and absent toxicity (cell viability higher than a threshold of 2x plate median of standard deviations). Tertiary screen hits were scored by products of SSMD values from individual siRNAs with SSMD < =−1.645.

## Additional Information

**How to cite this article**: Lupberger, J. *et al.* PI4K-beta and MKNK1 are regulators of hepatitis C virus IRES-dependent translation. *Sci. Rep.* **5**, 13344; doi: 10.1038/srep13344 (2015).

## Supplementary Material

Supplementary Information
