# Supplementary material for: PI4K-beta and MKNK1 are regulators of hepatitis C virus IRES-dependent translation
Source: Sci Rep. 2015 Sep 1;5:13344. doi: 10.1038/srep13344 (PMC4555030; doi:10.1038/srep13344)
Supplement: Supplementary Information [file srep13344-s1.pdf]

PI4K-beta and MKNK1 are regulators of hepatitis C virus IRES translation

Joachim Lupberger[1,2], Claudia Casanova[3], Benoit Fischer[4], Amelie Weiss[4], Isabel Fofana[1,2], Nelly Fontaine[1,2], Toshinobu Fujiwara[5], Mickael Renaud[4], Arnaud Kopp[4], Catherine Schuster[1,2], Laurent Brino[4], Thomas F. Baumert[1,2,6] and Christian Thoma[3].

Supplementary Information

Supplementary table S1: High-throughput screening results of the primary screen. Impact of target-specific siRNA silencing on IRES versus cap-dependent translation in Huh7.5 cells is compared as normalized ratios of relative light units of *firefly* (IRES) / *renilla* (cap) luciferase activities. (Mean ± sd from one experiment with three independent replicates on parallel plates). Screening results are scored by Z-values. Hits (Z-score <-1.28) are highlighted in bold and green. (§) Gene re-screened in a second experiment (technical failure of one plate in the first screen).

GeneID	norm_exp1	norm_exp2	norm_exp3	m	sd	Z-score
**CSNK2B**	0.718	0.572	0.610	0.633	0.076	-2.80
**ADK**	0.670	0.758	0.583	0.670	0.088	-2.51
**LTK**	0.614	0.720	0.775	0.703	0.082	-2.28
**ITPKA**	0.754	0.567	0.849	0.723	0.143	-2.14
**GK2**	0.641	0.814	0.716	0.724	0.087	-2.11
**PTPRN**	0.713	0.722	0.737	0.724	0.012	-2.11
**PIM1**	0.806	0.583	0.881	0.757	0.155	-1.88
**MGC75495**	0.708	0.762	0.798	0.756	0.045	-1.87
**PCTK3**	0.716	0.765	0.790	0.757	0.038	-1.86
**PIK4CB**	0.701	0.734	0.840	0.758	0.073	-1.86
**CDK7**	0.801	0.800	0.698	0.766	0.059	-1.77
**PTPN12**	0.897	0.721	0.687	0.768	0.113	-1.76
**PTPN2**	0.813	0.814	0.682	0.770	0.076	-1.75

**PDK4**	0.652	0.884	0.783	0.773	0.116	-1.74
**MKNK1**	0.693	0.837	0.794	0.775	0.074	-1.73
**DGKZ**	0.721	0.849	0.755	0.775	0.066	-1.72
**MINPP1**	0.713	0.876	0.742	0.777	0.087	-1.70
**CDK8**	0.926	0.782	0.619	0.776	0.154	-1.69
**GRK4**	0.782	0.793	0.766	0.780	0.014	-1.68
**NTRK1**	0.777	0.775	0.799	0.784	0.013	-1.66
**MAP3K4**	0.796	0.867	0.686	0.783	0.091	-1.64
**MAP3K12**	0.804	0.797	0.761	0.787	0.023	-1.62
**MST1R**	0.755	0.752	0.867	0.791	0.066	-1.61
**ROR1**	0.819	0.784	0.769	0.791	0.026	-1.60
**ALPI**	0.930	0.804	0.631	0.788	0.150	-1.59
**CSK**	0.759	0.869	0.742	0.790	0.069	-1.59
**KHK**	0.768	0.828	0.787	0.794	0.031	-1.57
**CDC42BPA**	0.765	0.750	0.877	0.797	0.069	-1.56
**PTPRS**	0.763	0.775	0.852	0.797	0.048	-1.56
**CDK4**	0.752	0.889	0.763	0.801	0.076	-1.51
**CIB3**	0.943	0.728	0.730	0.800	0.124	-1.51
**PFKFB4**	0.803	0.844	0.757	0.801	0.044	-1.51
**ULK1**	0.780	0.848	0.788	0.805	0.037	-1.49
**CDKN2B**	0.915	0.794	0.727	0.812	0.095	-1.42
**PFKM** (§)	0.750	0.677	0.750	0.726	0.042	-1.41
**GALK2**	0.736	0.951	0.760	0.816	0.118	-1.40
**STK25**	0.831	0.802	0.817	0.817	0.015	-1.40
**TYROBP**	0.760	0.802	0.903	0.822	0.074	-1.38
**PPP1R12C**	0.790	0.783	0.897	0.823	0.064	-1.36
**NYD-SP25**	0.686	0.760	1.044	0.830	0.189	-1.33
**PRKAA2**	0.867	0.849	0.758	0.825	0.058	-1.33
**MTMR12**	0.898	0.910	0.665	0.824	0.138	-1.32
**PTPLA**	0.976	0.769	0.732	0.826	0.132	-1.32
**CKMT2**	1.034	0.667	0.784	0.828	0.187	-1.31
**EPHA5**	0.824	0.814	0.858	0.832	0.023	-1.29
PPP2R4	0.846	0.770	0.885	0.834	0.058	-1.28
IMPA1	0.820	0.968	0.708	0.832	0.130	-1.26
PPP1CA	0.902	0.843	0.761	0.835	0.071	-1.25
PPM1G	0.805	0.933	0.769	0.836	0.086	-1.24
PRKAA1	0.814	0.734	0.982	0.843	0.127	-1.22
PKN1 (§)	0.849	0.821	0.650	0.773	0.108	-1.22
EPHA7	0.906	0.896	0.716	0.839	0.107	-1.21
IHPK3	0.914	0.768	0.843	0.842	0.073	-1.21
LIMK2	0.894	0.762	0.871	0.842	0.071	-1.21
WNK2	0.817	0.826	0.884	0.842	0.036	-1.21

CDKN2D	0.881	0.721	0.935	0.846	0.111	-1.20
MTMR6	0.864	0.822	0.845	0.844	0.021	-1.19
PDK3	0.877	0.790	0.867	0.845	0.048	-1.19
PTK7 (§)	0.740	0.744	0.850	0.778	0.062	-1.19
PRKCD	0.773	0.844	0.925	0.847	0.076	-1.18
ADCK4	0.769	0.785	0.996	0.850	0.127	-1.17
MKNK2	0.875	0.884	0.775	0.845	0.061	-1.17
IKBKB	0.900	0.829	0.812	0.847	0.047	-1.16
PRKY	0.873	0.828	0.842	0.848	0.023	-1.16
PTPN14	0.808	0.828	0.913	0.850	0.056	-1.16
PFKFB2	0.803	0.879	0.870	0.851	0.042	-1.15
PTPN22	0.864	0.846	0.838	0.849	0.013	-1.15
SNAP23	0.914	0.864	0.770	0.849	0.073	-1.14
PHKB	0.809	0.873	0.876	0.853	0.038	-1.13
CALM2	0.905	0.915	0.735	0.852	0.101	-1.11
CSNK1G1	0.854	0.840	0.870	0.855	0.015	-1.11
PPP2R5C	0.820	0.845	0.903	0.856	0.043	-1.11
PRKAG3	0.826	0.984	0.747	0.852	0.121	-1.11
PTPRG	0.897	0.793	0.880	0.857	0.056	-1.10
PIK3C2A	0.955	0.665	0.958	0.859	0.168	-1.09
ROR2	0.845	0.897	0.832	0.858	0.034	-1.08
AKT1	0.795	0.842	0.957	0.865	0.083	-1.05
PTPN13	0.860	0.838	0.896	0.865	0.029	-1.04
FGFR1	0.810	0.876	0.915	0.867	0.053	-1.03
AKAP13	0.966	0.808	0.823	0.866	0.087	-1.02
PLAU	0.981	0.715	0.909	0.868	0.138	-1.02
RIOK2	0.895	0.766	0.942	0.868	0.091	-1.02
JAK1	0.863	0.878	0.863	0.868	0.009	-1.01
PANK2	0.834	0.884	0.884	0.867	0.029	-1.01
NEK3	0.787	0.878	0.958	0.874	0.086	-0.97
PPP1R14D	0.922	0.916	0.784	0.874	0.078	-0.95
SPHK2	1.000	0.818	0.805	0.874	0.109	-0.95
DUSP6	0.832	0.873	0.930	0.878	0.049	-0.94
PPP3R1	0.846	0.904	0.882	0.877	0.029	-0.94
SGPP1	0.888	0.924	0.816	0.876	0.055	-0.94
NRBP	0.872	0.834	0.938	0.881	0.053	-0.92
PPP1R3C	0.805	0.904	0.933	0.881	0.067	-0.92
PPP2R1A	0.927	0.840	0.872	0.880	0.044	-0.92
GRK6	0.918	0.860	0.863	0.880	0.033	-0.91
MAP3K11	0.787	0.865	1.000	0.884	0.108	-0.91
MGC4796	0.895	0.816	0.935	0.882	0.061	-0.91
PTPN3	0.888	0.946	0.806	0.880	0.070	-0.91

PDK1 (§)	1.028	0.710	0.688	0.809	0.190	-0.91
FN3KRP	0.839	0.830	0.986	0.885	0.088	-0.90
PDIK1L	0.911	0.857	0.877	0.882	0.027	-0.90
PTPN20B	0.847	0.882	0.921	0.883	0.037	-0.90
STK36	0.833	0.895	0.922	0.883	0.046	-0.90
EIF2AK2 (§)	0.877	0.872	0.750	0.833	0.072	-0.90
CDK9	0.869	1.000	0.777	0.882	0.112	-0.89
DGKI	0.851	0.947	0.851	0.883	0.055	-0.89
PTPRR	0.841	0.929	0.880	0.883	0.044	-0.89
CAMK2G	0.881	0.921	0.849	0.884	0.036	-0.88
OXSR1	0.921	0.820	0.913	0.885	0.056	-0.88
CDKN1C	0.891	0.902	0.866	0.886	0.018	-0.87
JAK3	0.838	0.972	0.844	0.885	0.076	-0.87
AK2	0.847	0.935	0.887	0.890	0.044	-0.85
CKMT1	0.994	0.964	0.698	0.885	0.163	-0.85
STK24	0.944	0.872	0.852	0.889	0.048	-0.84
PRKACA (§)	0.685	0.872	0.975	0.844	0.147	-0.84
DOK1	1.000	0.848	0.823	0.890	0.096	-0.83
PRKAG1	0.885	0.854	0.940	0.893	0.044	-0.83
NYD-SP25	0.902	0.844	0.935	0.894	0.046	-0.82
SNARK	0.828	0.884	0.972	0.895	0.073	-0.82
CDK10	0.873	0.806	1.010	0.896	0.104	-0.81
CSNK2A2	0.949	0.727	1.014	0.897	0.150	-0.81
ILK	0.802	0.945	0.937	0.895	0.080	-0.81
LOC55971	0.799	0.909	0.980	0.896	0.091	-0.81
PAK1	0.822	0.988	0.870	0.893	0.085	-0.81
DGKD	0.916	0.843	0.929	0.896	0.046	-0.80
FUK	0.857	0.975	0.851	0.894	0.070	-0.80
MAP4K5	0.902	0.860	0.927	0.896	0.034	-0.80
PIK4CA	0.898	0.867	0.923	0.896	0.028	-0.80
NRGN (§)	0.833	1.032	0.719	0.861	0.158	-0.80
FLT4	0.923	0.848	0.922	0.898	0.043	-0.79
CIB2	0.933	0.892	0.868	0.898	0.033	-0.78
MAPK14	1.000	0.776	0.921	0.899	0.114	-0.78
PIK3CA	0.924	0.855	0.917	0.899	0.038	-0.78
PIP5K2A	0.879	0.855	0.964	0.899	0.057	-0.78
PPFIA2	0.910	0.910	0.871	0.897	0.023	-0.78
LRRK2 (§)	0.722	0.968	0.906	0.865	0.128	-0.78
CRK7	0.976	0.951	0.764	0.897	0.116	-0.77
KIT	0.648	1.048	1.007	0.901	0.220	-0.77
PI4K2B	0.799	0.893	1.012	0.901	0.107	-0.77
PPP1R12B	0.891	0.892	0.918	0.900	0.015	-0.76

STK22C	0.870	0.967	0.861	0.899	0.059	-0.76
ZC3HC1	0.839	0.914	0.952	0.902	0.058	-0.76
ACPP	0.922	0.997	0.785	0.901	0.108	-0.74
CASK	0.911	1.000	0.794	0.902	0.103	-0.74
PKIB	0.885	0.896	0.935	0.905	0.026	-0.73
PRKAR1A	0.820	0.896	1.003	0.906	0.092	-0.73
DGKH (§)	0.722	0.839	1.031	0.864	0.156	-0.73
AKAP8	0.893	0.886	0.939	0.906	0.029	-0.72
CSNK1G2	0.926	0.952	0.835	0.904	0.061	-0.72
FYN	1.003	0.695	1.026	0.908	0.185	-0.72
SORCS3	0.955	0.802	0.965	0.907	0.091	-0.72
LOC283155 (§)	0.889	0.806	0.875	0.857	0.044	-0.72
NEK7 (§)	0.778	0.968	0.875	0.874	0.095	-0.72
HK2	1.210	0.634	0.878	0.907	0.289	-0.71
PRKD2	0.909	0.847	0.969	0.908	0.061	-0.71
MAP2K5	0.909	0.906	0.909	0.908	0.002	-0.70
PTPN4	0.942	0.880	0.908	0.910	0.031	-0.69
PPM1L	0.912	0.898	0.926	0.912	0.014	-0.68
TIE1	0.961	0.930	0.839	0.910	0.063	-0.68
PKIA (§)	0.904	0.846	0.875	0.875	0.029	-0.67
CDC42BPB	0.966	0.854	0.921	0.914	0.056	-0.66
CHUK	0.847	0.908	0.990	0.915	0.072	-0.66
PIK3CG	1.013	0.871	0.860	0.915	0.085	-0.64
RPS6KA5	0.848	0.874	1.035	0.919	0.101	-0.64
PPP2R5A	0.795	1.050	0.908	0.918	0.128	-0.63
ROCK1 (§)	0.877	0.821	0.950	0.883	0.065	-0.63
CDK3	0.803	0.974	0.983	0.920	0.101	-0.62
MAPK11	0.850	1.042	0.862	0.918	0.108	-0.62
SLK	0.994	0.958	0.797	0.916	0.105	-0.62
ITPKC	0.965	0.972	0.819	0.919	0.086	-0.61
PAK3	0.911	0.931	0.917	0.920	0.010	-0.61
PTPRH	0.919	0.870	0.976	0.922	0.053	-0.61
MINK1	0.965	0.883	0.922	0.923	0.041	-0.59
PHPT1	0.898	0.970	0.897	0.922	0.042	-0.59
PRKG1	0.944	0.911	0.914	0.923	0.018	-0.59
NME5	0.883	0.872	1.026	0.927	0.086	-0.58
PPP1CB	0.907	0.931	0.935	0.924	0.015	-0.58
CKB	0.926	0.976	0.871	0.924	0.053	-0.57
PTPRD	0.976	0.763	1.046	0.928	0.147	-0.57
PTPRE	0.858	0.870	1.058	0.929	0.112	-0.57
GUK1 (§)	0.917	0.871	0.875	0.888	0.025	-0.57
STK11	0.916	0.854	1.015	0.928	0.081	-0.56

VRK3	0.927	0.912	0.944	0.928	0.016	-0.56
CaMKIINalpha	0.811	0.884	1.100	0.932	0.150	-0.55
ILKAP	0.918	0.987	0.874	0.926	0.057	-0.55
NTRK3	0.884	1.041	0.853	0.926	0.101	-0.55
BRAF	0.905	0.928	0.955	0.929	0.025	-0.54
PTPRM	0.898	1.030	0.856	0.928	0.091	-0.54
WEE1	0.848	0.973	0.970	0.930	0.071	-0.54
AAK1	0.960	0.971	0.857	0.929	0.063	-0.53
FRK	0.937	1.014	0.837	0.929	0.089	-0.53
CIT	0.955	0.970	0.868	0.931	0.055	-0.52
IKBKE	0.961	0.940	0.894	0.932	0.034	-0.52
STK16	0.933	1.012	0.845	0.930	0.084	-0.52
AK1	0.956	0.935	0.907	0.933	0.025	-0.51
CDC2	0.898	0.945	0.957	0.933	0.031	-0.51
DAPK1	0.954	1.006	0.834	0.931	0.088	-0.51
LOC392265	0.995	0.866	0.941	0.934	0.065	-0.51
MAGI1	1.065	0.815	0.920	0.933	0.126	-0.51
CAMKK1	0.870	0.884	1.060	0.938	0.106	-0.49
EIF2AK4	0.953	0.990	0.861	0.935	0.066	-0.49
PPP1R11	0.958	0.997	0.845	0.933	0.079	-0.49
PPP1R1A	0.977	0.926	0.901	0.935	0.039	-0.49
LOC390975 (§)	1.083	0.806	0.781	0.890	0.168	-0.49
AKIP	0.972	0.952	0.886	0.937	0.045	-0.48
CDK2	0.839	0.935	1.045	0.940	0.103	-0.48
CTDSPL	0.981	0.941	0.887	0.936	0.047	-0.48
FCRL2	0.912	0.872	1.034	0.939	0.084	-0.48
PDK2	0.949	0.977	0.882	0.936	0.049	-0.48
PHKG2	0.923	0.959	0.930	0.937	0.019	-0.48
PIP5K2C	0.882	0.959	0.972	0.938	0.049	-0.48
SIRPA	0.900	0.975	0.939	0.938	0.038	-0.48
PRKCA (§)	0.959	0.974	0.800	0.911	0.096	-0.48
FLJ14800	0.909	0.936	0.971	0.939	0.031	-0.47
FLT3	1.049	0.924	0.837	0.937	0.107	-0.47
NME2	0.929	0.909	0.979	0.939	0.036	-0.47
PIP5K1B	0.898	0.997	0.919	0.938	0.052	-0.47
MAPK3 (§)	0.767	0.974	1.000	0.914	0.128	-0.47
PFKL	0.975	0.931	0.911	0.939	0.033	-0.46
PHOSPHO1	0.888	0.987	0.945	0.940	0.050	-0.46
PRKCG	0.938	0.839	1.049	0.942	0.105	-0.46
PTPRZ1	0.919	0.876	1.031	0.942	0.080	-0.46
PTK6 (§)	1.041	0.897	0.800	0.913	0.121	-0.46
SKP1 (§)	1.041	0.846	0.850	0.912	0.111	-0.46

STK35 (§)	0.861	1.032	0.875	0.923	0.095	-0.46
LOC400927	1.039	0.992	0.783	0.938	0.136	-0.45
PTPN21	0.970	0.983	0.866	0.940	0.064	-0.45
LOC285940 (§)	0.972	0.839	0.906	0.906	0.067	-0.45
SRPK2 (§)	1.123	0.872	0.750	0.915	0.190	-0.45
ARAF	1.015	1.000	0.804	0.940	0.118	-0.44
C21orf124	0.751	1.074	1.004	0.943	0.170	-0.44
GRK7	0.932	0.880	1.022	0.945	0.072	-0.44
MELK	0.994	0.901	0.933	0.943	0.047	-0.44
NEK9	0.853	0.936	1.043	0.944	0.095	-0.44
STK6	0.947	0.997	0.879	0.941	0.059	-0.44
PRKACG (§)	0.658	0.974	1.125	0.919	0.238	-0.44
GK	0.979	0.903	0.950	0.944	0.038	-0.43
PPP1R9A	0.936	1.006	0.887	0.943	0.060	-0.43
YES1	0.978	0.955	0.894	0.942	0.043	-0.43
HIPK4 (§)	0.917	0.613	1.156	0.895	0.272	-0.43
MAPK10 (§)	0.959	0.846	0.950	0.918	0.063	-0.43
LOC400301	0.878	0.986	0.972	0.945	0.059	-0.42
PPP1R12A	0.957	0.933	0.944	0.945	0.012	-0.42
PPP2CA	1.051	0.931	0.851	0.944	0.101	-0.41
Sharpin	0.799	0.818	1.243	0.953	0.251	-0.41
ADCK2	0.895	1.000	0.949	0.948	0.053	-0.40
AKAP12	0.977	0.901	0.968	0.949	0.042	-0.40
MTMR3	0.946	0.983	0.912	0.947	0.036	-0.40
PLK3	1.034	0.921	0.885	0.947	0.078	-0.40
PPP2R1B	0.922	0.886	1.041	0.950	0.081	-0.40
PTPN7	0.881	1.096	0.862	0.946	0.130	-0.40
RPS6KC1	0.951	0.932	0.963	0.949	0.016	-0.40
ERK8 (§)	1.028	0.871	0.844	0.914	0.099	-0.40
IRAK1 (§)	0.833	0.871	1.062	0.922	0.123	-0.40
LOC391295 (§)	0.944	0.871	0.938	0.918	0.041	-0.40
PPP1R10	0.958	0.926	0.963	0.949	0.020	-0.39
SUMO1P3	0.979	0.940	0.926	0.948	0.027	-0.39
LIMK1	0.960	1.000	0.886	0.949	0.058	-0.38
MAPK6	0.926	0.880	1.049	0.952	0.087	-0.38
PTPRC	1.018	1.023	0.799	0.947	0.128	-0.38
ALK	0.960	0.964	0.928	0.951	0.020	-0.37
HCK	0.923	1.041	0.887	0.950	0.081	-0.37
ITPKB	1.076	0.939	0.834	0.950	0.121	-0.37
MAPK8IP1	0.937	1.058	0.851	0.949	0.104	-0.37
STYK1	1.000	0.858	1.000	0.953	0.082	-0.37
MAPK4 (§)	1.068	0.974	0.750	0.931	0.163	-0.37

PPP2R3B	0.958	1.037	0.856	0.950	0.091	-0.36
PPP6C	1.038	0.968	0.846	0.951	0.097	-0.36
ASK	0.931	0.959	0.974	0.955	0.022	-0.35
PIP5K1A	0.929	0.937	1.000	0.955	0.039	-0.35
MAPK8	0.832	1.031	1.008	0.957	0.109	-0.34
TENC1	0.988	0.963	0.912	0.954	0.039	-0.34
XYLB	0.854	0.988	1.029	0.957	0.092	-0.34
CDKL3	0.982	0.977	0.907	0.955	0.042	-0.33
PPP2R5B	1.033	0.915	0.918	0.955	0.067	-0.33
STK38L	0.833	1.041	1.000	0.958	0.110	-0.33
PRKAR2B (§)	1.041	0.769	1.000	0.937	0.147	-0.33
INPP5F	0.936	0.959	0.983	0.959	0.024	-0.32
PRKRA	1.039	1.000	0.826	0.955	0.113	-0.32
TRIO	0.899	0.959	1.019	0.959	0.060	-0.32
AK3L1	0.946	1.098	0.825	0.956	0.137	-0.31
CHEK1	0.898	0.974	1.010	0.961	0.057	-0.31
LPPR2	0.955	1.023	0.897	0.958	0.063	-0.31
MAPK7	0.950	0.948	0.982	0.960	0.019	-0.31
PIK3C2B	1.013	0.947	0.916	0.959	0.050	-0.31
PLK1	0.923	1.081	0.869	0.958	0.110	-0.31
DUSP21	0.965	0.885	1.036	0.962	0.076	-0.30
PPAP2C	0.897	1.041	0.942	0.960	0.074	-0.30
CDC25B	0.992	0.962	0.928	0.961	0.032	-0.29
CHKA	0.920	0.948	1.024	0.964	0.054	-0.29
PPP1R1B	0.979	0.925	0.983	0.962	0.032	-0.29
LOC391533 (§)	0.861	1.032	0.969	0.954	0.086	-0.29
EGFR	0.930	1.000	0.959	0.963	0.035	-0.28
PDXK	0.910	0.991	0.990	0.964	0.046	-0.28
PPP1R3B	0.906	0.992	0.994	0.964	0.050	-0.28
PTK9 (§)	0.795	1.077	0.975	0.949	0.143	-0.28
CDKN2C	0.909	1.000	0.986	0.965	0.049	-0.27
PPP3CA	0.832	1.000	1.070	0.967	0.122	-0.27
RIPK5	0.915	0.971	1.012	0.966	0.049	-0.27
TK2	0.899	0.953	1.045	0.966	0.074	-0.27
TRAD	0.961	1.000	0.932	0.964	0.034	-0.27
PRKX (§)	1.342	0.718	0.775	0.945	0.345	-0.27
DGKE	0.955	1.017	0.923	0.965	0.048	-0.26
ETNK2	0.878	1.114	0.901	0.964	0.130	-0.26
MARK2	0.991	0.988	0.915	0.965	0.043	-0.26
MGC40579	0.992	0.880	1.033	0.968	0.079	-0.26
VRK2	0.910	0.961	1.030	0.967	0.060	-0.26
HIPK1 (§)	0.944	0.935	0.969	0.949	0.018	-0.26

DGKG	0.944	1.000	0.957	0.967	0.029	-0.25
EPHB3	1.035	0.834	1.035	0.968	0.116	-0.25
NAGK	0.944	1.028	0.928	0.967	0.054	-0.25
LRRK1	0.958	0.922	1.029	0.970	0.054	-0.24
MAP2K1IP1	1.034	0.925	0.944	0.968	0.058	-0.24
MTMR4	1.081	0.964	0.852	0.966	0.115	-0.24
PIK3R4	0.921	0.921	1.072	0.971	0.087	-0.24
TPTE	0.946	0.977	0.985	0.969	0.021	-0.24
PRKCI (§)	1.014	1.026	0.825	0.955	0.113	-0.24
ADCK1	0.853	0.965	1.101	0.973	0.124	-0.23
G6PC3	0.947	0.994	0.971	0.971	0.024	-0.23
AURKC	0.983	0.890	1.045	0.973	0.078	-0.22
CHKB	0.964	0.967	0.990	0.974	0.014	-0.21
DYRK2	1.073	1.035	0.800	0.969	0.148	-0.21
MAP3K9	0.975	0.977	0.964	0.972	0.007	-0.21
PIP5K2B	0.879	0.997	1.045	0.974	0.085	-0.21
PPP4R1	0.930	1.000	0.990	0.973	0.038	-0.21
STK22B	0.997	0.984	0.934	0.972	0.033	-0.21
MAP2K3	0.914	0.974	1.039	0.976	0.063	-0.20
NME6	0.961	1.036	0.920	0.972	0.059	-0.20
WDSAM1	1.065	0.916	0.938	0.973	0.080	-0.20
DYRK1B	1.039	0.913	0.974	0.975	0.063	-0.19
PACSIN1	1.043	0.951	0.932	0.975	0.059	-0.18
MAP2K1 (§)	1.096	1.000	0.800	0.965	0.151	-0.18
GNE	1.000	1.018	0.915	0.978	0.055	-0.16
PPP1R3A	1.053	0.933	0.949	0.978	0.065	-0.16
PPP1R7	0.907	0.936	1.104	0.982	0.106	-0.16
PTPN23	0.994	0.963	0.979	0.979	0.016	-0.16
TESK2	0.960	0.994	0.985	0.980	0.018	-0.16
DYRK3	0.905	1.134	0.897	0.979	0.135	-0.15
PDLIM5	1.011	0.977	0.950	0.979	0.031	-0.15
DGUOK	1.007	0.841	1.106	0.985	0.134	-0.14
EPHB1	0.960	0.988	0.996	0.981	0.019	-0.14
PPM1D	0.897	1.053	0.997	0.982	0.079	-0.14
STK31	0.976	0.893	1.084	0.984	0.096	-0.14
EIF2AK3	0.897	0.930	1.131	0.986	0.127	-0.13
HIPK2	1.079	0.977	0.888	0.981	0.096	-0.13
HSPB8	0.946	0.997	1.006	0.983	0.032	-0.13
LOC91807	0.979	1.085	0.877	0.980	0.104	-0.13
RNGTT	0.880	1.024	1.052	0.985	0.092	-0.12
WNK3	1.018	0.992	0.940	0.983	0.040	-0.12
MARCKS (§)	1.000	1.194	0.781	0.992	0.207	-0.12

PPP1R3F (§)	0.972	1.032	0.938	0.981	0.048	-0.12
STK3 (§)	0.932	1.154	0.850	0.979	0.157	-0.12
GALK1	0.997	1.012	0.945	0.985	0.035	-0.11
MAP3K7	0.983	1.012	0.961	0.985	0.026	-0.11
PRKCQ	1.062	0.995	0.894	0.984	0.085	-0.11
PRKD3	0.982	0.814	1.174	0.990	0.180	-0.11
RPS6KA6	1.006	0.945	1.006	0.986	0.035	-0.11
SGK2	1.006	1.000	0.950	0.985	0.031	-0.11
STK23	1.012	1.049	0.888	0.983	0.084	-0.11
DCAMKL1	0.931	0.906	1.131	0.989	0.123	-0.10
PIK3R3	0.929	0.896	1.146	0.990	0.136	-0.10
PPFIA1	0.952	0.997	1.015	0.988	0.032	-0.10
PTPN1	1.028	1.098	0.823	0.983	0.143	-0.10
UHMK1	0.992	0.964	1.007	0.988	0.022	-0.10
UMP-CMPK	0.866	0.847	1.267	0.993	0.237	-0.10
ARHGAP26	1.006	1.029	0.925	0.987	0.055	-0.09
BMPR2	1.190	0.974	0.790	0.985	0.200	-0.09
BRSK1	1.041	1.008	0.908	0.986	0.069	-0.09
FES	0.937	1.028	1.000	0.988	0.047	-0.09
PPP4C	0.958	1.000	1.008	0.989	0.027	-0.09
NEK4 (§)	1.096	0.974	0.875	0.982	0.111	-0.09
AKAP8L	0.976	0.853	1.149	0.993	0.149	-0.08
JAK2	0.918	0.945	1.114	0.992	0.106	-0.08
M-RIP	1.036	0.935	0.997	0.989	0.051	-0.08
PPM1E	1.086	0.982	0.893	0.987	0.097	-0.08
BMX (§)	0.944	1.065	0.969	0.993	0.064	-0.08
PRPS1L1 (§)	1.028	0.903	1.000	0.977	0.066	-0.08
ROS1 (§)	0.932	0.974	1.050	0.985	0.060	-0.08
ALPK1	0.979	1.000	0.993	0.991	0.011	-0.07
CINP	1.012	1.101	0.851	0.988	0.127	-0.07
FLJ13052	1.063	0.979	0.928	0.990	0.068	-0.07
SBF1	1.024	0.963	0.985	0.991	0.031	-0.07
STK10	0.929	1.045	1.000	0.991	0.058	-0.07
STK38	1.080	0.924	0.968	0.991	0.080	-0.07
TLK2	0.943	0.994	1.038	0.992	0.048	-0.07
MAPK8IP3	0.940	1.067	0.970	0.992	0.066	-0.06
PPP1R1C	1.051	0.985	0.937	0.991	0.057	-0.06
TEK	0.966	0.973	1.040	0.993	0.041	-0.06
ACVR2 (§)	0.944	0.839	1.156	0.980	0.161	-0.06
GKAP1	1.042	0.972	0.964	0.993	0.043	-0.05
MASTL	1.105	0.957	0.913	0.992	0.101	-0.05
MERTK	0.826	1.144	1.010	0.993	0.160	-0.05

UCK2	1.017	0.843	1.129	0.996	0.144	-0.05
IRAK4	0.994	1.003	0.988	0.995	0.008	-0.04
MATK	0.916	1.034	1.035	0.995	0.068	-0.04
PFTK1	1.000	1.122	0.853	0.992	0.135	-0.04
RPS6KL1	1.049	0.915	1.022	0.995	0.071	-0.04
ZAP70	1.003	1.051	0.924	0.993	0.064	-0.04
ACVR2B (§)	0.889	1.129	1.000	1.006	0.120	-0.04
CSNK1D	0.927	1.033	1.031	0.997	0.061	-0.03
DMPK	1.113	0.931	0.943	0.996	0.102	-0.03
INPP5A	1.051	0.956	0.980	0.996	0.049	-0.03
PDPK1	1.200	0.765	1.028	0.998	0.219	-0.03
SNF1LK	1.005	0.961	1.022	0.996	0.031	-0.03
MARK1	1.025	1.000	0.964	0.996	0.031	-0.02
PACSIN3	0.959	0.984	1.054	0.999	0.049	-0.02
PIK3R5	1.085	1.029	0.870	0.995	0.112	-0.02
PPP1R2	1.008	0.985	1.000	0.998	0.012	-0.02
PPP2CB	0.952	1.026	1.015	0.998	0.040	-0.02
SKIP	1.000	0.972	1.022	0.998	0.025	-0.02
STK32B (§)	1.167	0.742	1.000	0.970	0.214	-0.02
INPP5D	1.098	0.936	0.958	0.997	0.088	-0.01
KIDINS220	1.047	1.041	0.900	0.996	0.083	-0.01
MAPKAPK3	1.039	1.029	0.923	0.997	0.064	-0.01
PPAP2A	1.060	0.990	0.943	0.998	0.059	-0.01
PPAP2B	1.058	1.000	0.935	0.998	0.062	-0.01
PTPRQ	0.882	1.060	1.057	1.000	0.102	-0.01
AKAP4	1.003	0.973	1.025	1.000	0.026	0.00
AKT3	0.893	0.976	1.138	1.002	0.125	0.00
FLT3LG	1.007	1.000	0.993	1.000	0.007	0.00
CLK3	0.994	1.030	0.978	1.001	0.027	0.01
LMTK2	1.017	0.874	1.119	1.003	0.123	0.01
LOC407835	1.016	1.048	0.935	1.000	0.058	0.01
PAK4	0.931	0.982	1.096	1.003	0.084	0.01
PAK6	0.953	1.017	1.036	1.002	0.043	0.01
ULK3	0.984	0.940	1.084	1.003	0.074	0.01
HK1	0.923	0.993	1.099	1.005	0.089	0.02
PINK1	1.105	0.965	0.935	1.002	0.091	0.02
PLK2	0.955	0.982	1.074	1.004	0.062	0.02
TYRO3	1.040	1.099	0.859	0.999	0.125	0.02
CALM3	0.956	1.059	0.997	1.004	0.052	0.03
TLK1	1.149	1.023	0.828	1.000	0.162	0.03
TNK1	1.015	1.051	0.939	1.002	0.057	0.03
TTK	0.997	0.973	1.045	1.005	0.037	0.03

GRK5 (§)	0.861	1.065	1.125	1.017	0.138	0.03
AK5	0.934	1.054	1.030	1.006	0.063	0.04
MAP4K3	0.861	1.093	1.066	1.007	0.127	0.04
MUSK	1.070	0.919	1.030	1.006	0.078	0.04
UCK1	0.923	1.092	1.000	1.005	0.085	0.04
MAPK12 (§)	0.849	1.282	0.900	1.010	0.237	0.04
ATM	1.036	0.895	1.093	1.008	0.102	0.05
CCRK	0.878	0.893	1.267	1.013	0.220	0.05
CDC42SE2	0.937	1.028	1.058	1.008	0.063	0.05
PDGFRB	1.006	1.023	0.988	1.006	0.018	0.05
PRKAB2	0.932	1.021	1.070	1.008	0.070	0.05
CERK	1.012	1.058	0.948	1.006	0.055	0.06
CTDSP2	0.908	0.982	1.141	1.010	0.119	0.06
EPS8L1	0.976	1.049	1.000	1.008	0.037	0.06
MAPKAPK5	0.954	0.973	1.101	1.009	0.080	0.06
PKMYT1	1.050	0.965	1.006	1.007	0.043	0.06
PPP3CC	0.953	1.118	0.946	1.006	0.097	0.06
STK29	1.164	0.973	0.879	1.005	0.145	0.06
STK33	0.959	0.876	1.203	1.013	0.170	0.06
AKAP11	1.163	0.916	0.945	1.008	0.135	0.07
CLK1	1.044	0.980	1.003	1.009	0.032	0.07
PIK3C3	1.045	0.867	1.124	1.012	0.132	0.07
PTPRF	0.724	1.189	1.120	1.011	0.251	0.07
NEK11	1.000	1.058	0.972	1.010	0.044	0.08
MAP2K7 (§)	0.849	1.103	1.100	1.017	0.146	0.08
TTBK2 (§)	0.972	1.097	1.000	1.023	0.066	0.08
IRAK1BP1	1.092	0.950	0.993	1.012	0.073	0.09
PLK4	0.876	1.144	1.016	1.012	0.134	0.09
PPP2R2C	1.103	1.029	0.896	1.009	0.105	0.09
PTPN5	1.088	0.970	0.977	1.012	0.066	0.09
RET	0.914	1.078	1.044	1.012	0.087	0.09
RIPK4	1.049	0.840	1.155	1.015	0.160	0.09
TAOK1	1.047	1.000	0.988	1.012	0.031	0.09
VRK1	1.017	1.000	1.019	1.012	0.010	0.09
BMPR1A	0.938	1.012	1.094	1.015	0.078	0.10
CAMK1	0.927	0.913	1.213	1.018	0.169	0.10
CAMK1G	1.118	1.074	0.837	1.010	0.151	0.10
ETNK1	1.000	0.967	1.076	1.014	0.056	0.10
GIT2	0.989	1.053	0.997	1.013	0.035	0.10
HGS	0.961	0.814	1.284	1.020	0.240	0.10
MAK	1.000	1.087	0.947	1.011	0.071	0.10
TBK1	1.003	1.022	1.012	1.012	0.010	0.10

PHKA1	1.019	0.988	1.036	1.014	0.024	0.11
SYK	0.849	0.860	1.355	1.021	0.289	0.11
CDKL1	1.017	1.006	1.026	1.016	0.010	0.12
HIPK3	0.828	1.029	1.201	1.019	0.187	0.12
PPP1CC	1.009	0.946	1.099	1.018	0.077	0.12
WNK1	0.979	1.007	1.065	1.017	0.044	0.12
INPP5B	0.925	1.078	1.048	1.017	0.081	0.13
NTRK2	1.097	0.947	1.007	1.017	0.075	0.13
STK11IP	0.968	1.078	1.004	1.017	0.056	0.13
PRKDC (§)	1.111	0.774	1.125	1.003	0.199	0.13
ABL1	1.080	1.059	0.907	1.015	0.094	0.14
PPP2R5E	0.942	1.201	0.903	1.015	0.162	0.14
CLK4	1.166	0.992	0.892	1.017	0.139	0.15
DUSP2	0.927	1.190	0.938	1.018	0.149	0.15
EEF2K	1.079	1.003	0.976	1.019	0.053	0.15
IKBKAP	1.108	1.099	0.838	1.015	0.153	0.15
NME4	0.981	1.081	0.994	1.019	0.054	0.15
SGKL	0.965	1.105	0.988	1.019	0.075	0.15
TAOK2 2	0.971	1.000	1.090	1.020	0.062	0.15
BCR (§)	0.889	1.226	1.031	1.049	0.169	0.15
PPM1B	1.053	1.003	1.005	1.020	0.028	0.16
ROCK2	1.034	0.982	1.048	1.021	0.035	0.16
TGFBR1	0.997	1.003	1.066	1.022	0.038	0.16
ULK2	1.006	1.000	1.061	1.022	0.034	0.16
STK39 (§)	1.056	1.194	0.875	1.042	0.160	0.16
MAST4	1.091	1.079	0.886	1.019	0.115	0.17
PTK9L	1.121	0.877	1.073	1.024	0.129	0.17
SKIP	1.114	0.988	0.962	1.021	0.081	0.17
AKAP14 (§)	0.917	1.000	1.188	1.035	0.139	0.17
CNKSR1	1.017	1.096	0.952	1.022	0.072	0.18
DUSP13	1.042	0.929	1.107	1.026	0.090	0.18
PHKA2	0.949	1.121	1.000	1.023	0.088	0.18
TAOK2 1	0.851	1.123	1.102	1.025	0.151	0.18
MOS	0.923	1.072	1.084	1.026	0.090	0.19
PPP1R3D	1.013	0.997	1.067	1.026	0.037	0.19
PPP2R2B	1.084	1.064	0.918	1.022	0.091	0.19
KIAA1804	1.049	1.035	0.993	1.026	0.029	0.20
MAP3K2	1.067	1.090	0.913	1.023	0.096	0.20
RIOK3	1.022	1.163	0.884	1.023	0.140	0.20
MAPK9 (§)	1.233	0.872	1.000	1.035	0.183	0.20
ALS2CR2	0.935	0.868	1.299	1.034	0.232	0.21
HUNK	0.953	1.016	1.120	1.030	0.084	0.21

SNF1LK2	1.054	1.067	0.958	1.026	0.060	0.21
TESK1	1.077	0.901	1.111	1.030	0.113	0.21
EIF2AK1	1.167	0.952	0.964	1.028	0.121	0.22
PRKCE	1.103	0.953	1.029	1.028	0.075	0.22
PSKH1	1.103	1.073	0.904	1.027	0.107	0.22
PTPN6	0.987	1.096	1.000	1.028	0.060	0.22
SMG1	1.047	0.946	1.096	1.030	0.076	0.22
PRKACB (§)	0.877	1.154	1.100	1.044	0.147	0.22
RPS6KA2 (§)	1.370	1.077	0.675	1.041	0.349	0.22
ITK	0.951	1.083	1.057	1.030	0.070	0.23
MAGI-3	1.016	1.045	1.028	1.030	0.015	0.23
MAPKAPK2	0.899	1.204	0.984	1.029	0.157	0.23
PFKP	1.013	1.116	0.958	1.029	0.080	0.23
PTP4A2	1.019	1.047	1.024	1.030	0.015	0.23
RIOK1	0.958	1.142	0.986	1.029	0.099	0.23
PRKRIR (§)	0.932	1.077	1.125	1.045	0.100	0.23
ADRBK2	1.029	0.837	1.244	1.037	0.204	0.24
APEG1	1.023	1.000	1.073	1.032	0.037	0.24
PCK1	1.045	1.041	1.007	1.031	0.021	0.24
PTPRB	1.246	1.010	0.825	1.027	0.211	0.24
CSNK1E	1.015	1.131	0.948	1.031	0.093	0.25
PRKCBP1	0.984	0.978	1.145	1.036	0.095	0.25
RIPK1	0.960	1.212	0.919	1.030	0.159	0.25
PRKG2 (§)	1.041	0.974	1.125	1.047	0.076	0.25
IKBKG	1.071	1.140	0.879	1.030	0.135	0.26
PANK4	0.927	0.899	1.292	1.039	0.219	0.26
PGK1	1.032	1.003	1.070	1.035	0.034	0.26
PTPDC1	1.024	1.038	1.042	1.035	0.009	0.26
RAGE	0.914	0.948	1.252	1.038	0.186	0.26
TEC	1.039	1.006	1.058	1.034	0.026	0.26
TNNI3K	0.982	1.114	1.006	1.034	0.070	0.26
CAMK4	0.796	1.033	1.292	1.040	0.248	0.27
DUSP10	1.000	0.997	1.113	1.037	0.066	0.27
PRKCABP	1.034	1.024	1.048	1.035	0.012	0.27
RBKS	0.958	0.929	1.232	1.040	0.167	0.27
DUSP3	0.991	1.103	1.014	1.036	0.059	0.28
PPP1R14C	1.009	1.060	1.040	1.036	0.026	0.28
PTPN11	1.051	1.010	1.048	1.036	0.023	0.28
DDR2	1.025	1.098	0.988	1.037	0.056	0.29
PASK	1.161	0.971	0.976	1.036	0.108	0.29
TNIK	1.180	0.908	1.024	1.037	0.136	0.29
CAMK1D	1.021	1.007	1.094	1.041	0.047	0.30

MAP3K13	1.000	0.994	1.131	1.042	0.077	0.30
NUCKS	0.870	1.033	1.227	1.043	0.179	0.30
PAK7	1.000	0.986	1.138	1.041	0.084	0.30
PKIG	1.241	1.018	0.845	1.035	0.199	0.30
PPP2R3A	0.967	1.093	1.059	1.040	0.065	0.30
GSK3B	1.162	0.938	1.021	1.040	0.113	0.31
ICK	1.163	0.958	0.997	1.039	0.109	0.31
MADD	1.071	1.104	0.939	1.038	0.087	0.31
PIM2	1.062	1.096	0.958	1.039	0.072	0.31
CAMKK2	1.023	1.164	0.933	1.040	0.116	0.32
CDKN3	1.165	0.915	1.046	1.042	0.125	0.32
PPM1M	1.059	1.019	1.049	1.042	0.021	0.32
TNK2	0.989	1.024	1.119	1.044	0.067	0.32
TP53RK	0.935	1.066	1.131	1.044	0.100	0.32
INPP4B	1.096	1.003	1.031	1.043	0.048	0.33
MTMR8	1.125	0.959	1.045	1.043	0.083	0.33
AXL	1.066	0.948	1.127	1.047	0.091	0.34
EPHA4	1.014	1.124	0.993	1.044	0.070	0.34
PPP2R2A	0.967	1.090	1.077	1.045	0.068	0.34
PRPF4B	1.022	1.017	1.097	1.045	0.045	0.34
TYK2	1.084	1.057	0.990	1.044	0.048	0.34
PSTPIP1	1.036	1.095	1.003	1.045	0.047	0.35
STK32A	1.097	1.036	1.001	1.045	0.049	0.35
TAOK3	0.927	0.971	1.253	1.050	0.177	0.35
NEK8 (§)	1.139	0.968	1.062	1.056	0.086	0.35
CDKL4	0.989	1.014	1.146	1.050	0.084	0.36
ERN1	1.003	1.061	1.077	1.047	0.039	0.36
MAP3K1	0.981	1.078	1.084	1.048	0.058	0.36
SRMS	1.056	1.219	0.852	1.042	0.184	0.36
STK4 (§)	1.151	1.026	1.025	1.067	0.072	0.36
PANK1	1.047	0.965	1.139	1.050	0.087	0.37
TPK1	1.022	0.984	1.145	1.050	0.084	0.37
AKAP10	1.112	1.030	1.003	1.048	0.057	0.38
ARK5	0.944	1.054	1.158	1.052	0.107	0.38
EPHB4	1.000	1.186	0.957	1.048	0.122	0.38
PPEF2	1.140	0.980	1.031	1.050	0.082	0.39
PTEN	1.037	1.059	1.059	1.052	0.013	0.39
PTPRV	1.118	1.015	1.017	1.050	0.059	0.39
PACSIN2	1.287	0.836	1.032	1.052	0.226	0.40
PRKCH	1.068	1.094	0.992	1.051	0.053	0.40
SSTK	1.041	1.025	1.100	1.055	0.040	0.41
AKAP3	0.966	1.099	1.102	1.056	0.078	0.42

CARKL	1.098	1.225	0.826	1.050	0.204	0.42
CHEK2	1.017	1.152	0.991	1.053	0.086	0.42
ENPP1	1.164	1.005	0.992	1.054	0.096	0.42
PBK	0.965	0.957	1.254	1.059	0.169	0.42
PPTC7	1.006	1.115	1.042	1.054	0.056	0.42
PTPN9	0.967	1.230	0.959	1.052	0.154	0.42
MAP3K3 (§)	1.222	0.935	1.031	1.063	0.146	0.42
ACVRL1	0.915	1.115	1.144	1.058	0.125	0.43
BCKDK	1.000	1.158	1.009	1.056	0.089	0.43
EPHA2	0.997	1.073	1.100	1.057	0.053	0.43
ITPK1	0.761	1.193	1.227	1.060	0.260	0.43
PTPRN2	0.930	1.166	1.072	1.056	0.119	0.43
ALS2CR7	1.049	1.078	1.043	1.057	0.019	0.44
PIP5K1C	1.091	1.107	0.969	1.056	0.075	0.44
PPP3CB	0.967	1.078	1.132	1.059	0.084	0.44
PRKCZ	1.068	0.974	1.138	1.060	0.082	0.44
PSKH2	1.006	1.033	1.139	1.059	0.070	0.44
PSTPIP2	1.070	1.095	1.003	1.056	0.048	0.44
RPS6KA1 (§)	1.315	0.897	1.025	1.079	0.214	0.44
ACVR1	0.949	1.020	1.216	1.062	0.138	0.45
EPHA8	1.222	1.134	0.804	1.053	0.220	0.45
MAP3K6 (§)	1.083	0.839	1.281	1.068	0.221	0.45
BMP2K	1.049	1.007	1.130	1.062	0.063	0.46
HK3	1.003	1.171	1.004	1.059	0.097	0.46
IHPK2	1.186	1.079	0.904	1.056	0.142	0.46
PPP1R14A	1.082	1.015	1.086	1.061	0.040	0.46
CSNK2A1	1.165	1.152	0.856	1.058	0.175	0.47
EPHA1	1.141	0.972	1.071	1.061	0.085	0.47
STK17B	1.112	0.952	1.125	1.063	0.096	0.47
TK1	1.089	0.988	1.110	1.062	0.065	0.47
AKAP1	1.123	1.157	0.897	1.059	0.141	0.48
IBTK	1.054	0.990	1.151	1.065	0.081	0.48
PPP1R9B	1.118	1.068	1.000	1.062	0.059	0.48
PTP4A3	1.108	0.990	1.093	1.064	0.064	0.48
T3JAM	1.136	1.083	0.964	1.061	0.088	0.48
MAPK13 (§)	0.795	1.128	1.350	1.091	0.279	0.48
DUSP14	1.131	1.024	1.038	1.064	0.058	0.49
NLK	1.030	1.036	1.130	1.065	0.056	0.49
STK17A	1.079	0.958	1.164	1.067	0.104	0.49
CSNK1A1L (§)	1.000	1.032	1.250	1.094	0.136	0.49
AKAP9	1.057	1.088	1.050	1.065	0.020	0.50
DAPK2	1.054	1.137	1.000	1.064	0.069	0.50

SH3KBP1	0.884	1.023	1.304	1.070	0.214	0.50
NEK2	1.000	1.053	1.154	1.069	0.078	0.51
NME7	1.148	1.149	0.892	1.063	0.148	0.51
PPP1R8	1.103	0.985	1.115	1.068	0.072	0.51
RPS6KA4	1.101	1.000	1.103	1.068	0.059	0.51
CKS2	1.000	1.222	0.976	1.066	0.136	0.52
DYRK1A	0.991	1.122	1.092	1.068	0.069	0.52
DYRK4	1.078	1.116	1.006	1.067	0.056	0.52
FASTK	1.149	1.000	1.055	1.068	0.075	0.52
MTMR2	1.042	1.084	1.077	1.068	0.023	0.52
NME3	1.096	0.965	1.148	1.070	0.094	0.52
STK19	1.071	1.128	1.000	1.066	0.064	0.52
AKAP5	0.983	1.070	1.160	1.071	0.089	0.53
GIT1	1.091	1.055	1.062	1.069	0.019	0.53
PIK3C2G	1.096	1.064	1.047	1.069	0.025	0.53
TXK	1.139	1.069	0.995	1.068	0.072	0.53
AKAP6	1.096	1.024	1.093	1.071	0.041	0.54
MST4	1.098	1.130	0.976	1.068	0.081	0.54
NJMU-R1	1.018	1.000	1.203	1.074	0.112	0.54
DUSP22	1.069	1.188	0.949	1.069	0.120	0.55
PMVK	1.135	1.126	0.945	1.069	0.107	0.55
MET	1.070	1.064	1.089	1.074	0.013	0.56
TTBK1	1.070	1.191	0.949	1.070	0.121	0.56
G6PC	1.129	1.079	1.010	1.073	0.060	0.57
PIK3AP1 (§)	1.278	0.935	1.062	1.092	0.173	0.58
KDR	1.035	1.110	1.085	1.077	0.038	0.59
MAP3K5	0.981	1.202	1.047	1.077	0.113	0.59
PCTK2	1.142	1.103	0.979	1.075	0.085	0.59
PTPN18	1.198	1.077	0.948	1.074	0.125	0.59
UGP2	1.034	1.116	1.084	1.078	0.041	0.59
DUSP11	1.070	1.030	1.141	1.080	0.056	0.60
LATS1	1.056	1.041	1.142	1.080	0.055	0.60
MAPKBP1	1.090	1.090	1.055	1.078	0.020	0.60
TGFBR2	1.164	1.039	1.035	1.079	0.073	0.61
CLK2	1.036	1.222	0.976	1.078	0.128	0.62
ENPP3	1.061	1.039	1.149	1.083	0.058	0.62
ANKK1	1.146	1.056	1.049	1.084	0.054	0.64
LHPP	1.088	1.159	1.003	1.083	0.078	0.65
PIB5PA	1.078	1.151	1.021	1.083	0.065	0.65
MARK3	1.116	1.110	1.028	1.085	0.049	0.66
CDC25A	1.173	1.078	1.008	1.086	0.083	0.67
SNRK	1.128	1.036	1.099	1.088	0.047	0.67

LOC161635 (§)	1.167	1.323	0.938	1.143	0.194	0.67
PDPR	1.019	1.142	1.107	1.089	0.063	0.68
PTPLB	1.071	1.121	1.074	1.089	0.028	0.68
PXK	0.978	1.073	1.229	1.093	0.127	0.69
ALPL	1.098	1.059	1.121	1.093	0.031	0.70
GAK	1.112	1.000	1.166	1.093	0.085	0.70
LOC375449	1.143	1.070	1.059	1.091	0.046	0.70
MAP4K1	1.195	1.076	0.997	1.089	0.100	0.70
PHKG1	1.226	0.997	1.049	1.091	0.120	0.70
PRKAR2A (§)	1.014	1.256	1.125	1.132	0.121	0.70
ERN2	1.080	1.193	1.003	1.092	0.096	0.72
PPP5C	1.043	1.172	1.067	1.094	0.069	0.72
PRSS7	1.204	0.964	1.117	1.095	0.122	0.72
ADCK5	1.219	0.975	1.094	1.096	0.122	0.73
FN3K	1.130	1.207	0.940	1.092	0.137	0.73
ZAK	1.091	1.105	1.090	1.095	0.008	0.73
PRKAR1B	1.392	1.000	0.883	1.092	0.267	0.74
GCK	1.155	1.055	1.085	1.098	0.051	0.75
MARK4	1.042	1.085	1.174	1.100	0.067	0.75
PPM1A	1.159	0.950	1.195	1.101	0.132	0.76
CDC25C	1.149	0.985	1.174	1.103	0.103	0.77
MAP3K8	1.216	1.261	0.806	1.094	0.251	0.77
DUSP16	1.094	1.140	1.068	1.101	0.036	0.78
PTK2B	1.094	1.104	1.107	1.102	0.007	0.78
NEK1	1.083	1.133	1.095	1.104	0.026	0.79
PRKCB1 (§)	1.041	1.154	1.250	1.148	0.105	0.79
RPS6KB2	1.056	1.078	1.184	1.106	0.068	0.80
SKP2	1.133	1.141	1.039	1.104	0.057	0.81
STK22D	1.124	1.223	0.964	1.104	0.131	0.82
PPP2R2D	0.991	1.263	1.069	1.108	0.140	0.83
DCAMKL2 (§)	1.222	1.194	1.062	1.159	0.085	0.83
MAP3K10	1.071	1.204	1.056	1.110	0.081	0.85
PPM1F	0.934	1.130	1.278	1.114	0.173	0.85
RPS6KA3	1.027	0.948	1.377	1.117	0.228	0.85
SGK1 (§)	0.849	1.359	1.275	1.161	0.273	0.85
DCK	1.082	1.134	1.122	1.113	0.027	0.86
PGK2	1.064	1.127	1.148	1.113	0.044	0.86
ERBB2	1.254	1.069	1.014	1.112	0.126	0.87
NEK6	1.178	1.216	0.945	1.113	0.147	0.89
LMTK3	0.923	0.950	1.506	1.126	0.329	0.90
MAP2K2	1.192	1.156	0.997	1.115	0.104	0.90
MAP2K4 (§)	1.151	1.051	1.300	1.167	0.125	0.90

TPTE2	1.124	1.089	1.146	1.120	0.029	0.91
CDC2L1	1.124	1.105	1.134	1.121	0.015	0.92
DUSP19	0.979	1.241	1.143	1.121	0.132	0.92
PTPRA	1.222	1.064	1.077	1.121	0.088	0.93
ADRBK1	1.255	1.059	1.052	1.122	0.115	0.94
AURKB	1.270	0.994	1.102	1.122	0.139	0.94
MAP3K7IP1	1.079	1.329	0.958	1.122	0.189	0.96
BUB1	1.199	1.133	1.043	1.125	0.078	0.97
CAMK2A	1.000	1.261	1.120	1.127	0.131	0.97
DUSP12	1.251	1.077	1.046	1.125	0.111	0.97
PPME1	1.226	1.077	1.079	1.127	0.085	0.98
PCTK1	1.219	1.066	1.105	1.130	0.080	1.00
SRPK1	1.086	1.365	0.930	1.127	0.220	1.00
MAP4K2 (§)	1.425	1.128	1.000	1.184	0.218	1.00
WNK4	1.124	1.223	1.044	1.130	0.090	1.01
AKAP7	1.180	1.281	0.926	1.129	0.183	1.02
MAP4K4	1.201	1.094	1.102	1.132	0.060	1.02
PDGFRA	1.134	1.023	1.254	1.137	0.116	1.03
PKN3	1.213	1.244	0.932	1.130	0.172	1.03
ALPPL2	1.145	1.088	1.177	1.137	0.045	1.04
IRAK3	1.264	1.078	1.061	1.134	0.113	1.04
KIAA1446	1.119	1.170	1.116	1.135	0.030	1.04
PRKAG2	1.205	1.187	1.006	1.133	0.110	1.04
ACP1	1.180	1.195	1.031	1.135	0.091	1.05
PPFIA3	1.120	1.171	1.119	1.137	0.030	1.05
AATK	1.219	1.162	1.029	1.137	0.098	1.06
FLJ10986	0.945	0.997	1.497	1.146	0.305	1.06
MYLK	1.102	1.046	1.278	1.142	0.121	1.06
PPM2C	1.114	1.197	1.103	1.138	0.051	1.06
CDK5R1	1.096	1.224	1.096	1.139	0.074	1.07
GSK3A	1.070	1.171	1.188	1.143	0.064	1.09
RFK	1.231	1.234	0.949	1.138	0.164	1.09
FBP1	1.205	1.038	1.195	1.146	0.094	1.11
ACP2	1.266	1.085	1.087	1.146	0.104	1.12
DDR1	1.170	1.079	1.194	1.148	0.061	1.12
DOLPP1	1.160	1.271	1.000	1.144	0.136	1.12
GRK1	0.973	1.505	0.945	1.141	0.316	1.12
LCK	1.198	0.939	1.314	1.150	0.192	1.12
PI4KII	1.196	1.092	1.152	1.147	0.052	1.12
ACP5	1.149	1.201	1.087	1.146	0.057	1.13
MAPK8IP2	1.098	1.127	1.224	1.150	0.066	1.13
PRKAB1	1.286	1.047	1.112	1.148	0.124	1.14

ACVR1B	1.263	1.065	1.120	1.149	0.102	1.15
ATR	1.285	1.118	1.045	1.149	0.123	1.16
IHPK1	1.172	1.070	1.219	1.154	0.076	1.16
KSR2 (§)	1.194	1.065	1.375	1.211	0.156	1.16
ULK4	1.199	1.251	1.000	1.150	0.132	1.17
LOC390777 (§)	1.139	1.194	1.344	1.226	0.106	1.17
INPP5E	1.184	1.278	0.989	1.150	0.147	1.18
MTMR9	1.118	1.053	1.303	1.158	0.130	1.18
CDKN1A (§)	1.472	1.161	1.000	1.211	0.240	1.18
CDKL5	1.238	1.236	0.980	1.151	0.148	1.19
PRKD1 (§)	0.986	1.410	1.275	1.224	0.217	1.19
PTPRK	1.198	1.104	1.180	1.161	0.050	1.22
PANK3	1.084	1.213	1.196	1.164	0.070	1.25
ACP6	1.036	1.231	1.232	1.166	0.113	1.26
BLK	1.159	1.055	1.288	1.167	0.117	1.26
CDK5R2	1.257	1.233	0.994	1.161	0.145	1.26
MAP3K7IP2	1.116	1.270	1.106	1.164	0.092	1.26
STK32C	1.135	1.176	1.186	1.166	0.027	1.26
AKT2	1.074	1.152	1.281	1.169	0.105	1.27
CDKL2	1.146	1.385	0.955	1.162	0.215	1.27
DGKA (§)	1.028	1.226	1.500	1.251	0.237	1.27
MAST2	1.006	1.329	1.168	1.168	0.162	1.28
PKM2	1.013	1.254	1.238	1.168	0.135	1.28
RIPK3	1.236	1.186	1.074	1.165	0.083	1.28
CAMK2D	1.250	1.127	1.129	1.169	0.070	1.30
PDP2	1.171	1.204	1.133	1.169	0.036	1.30
PNCK	1.243	1.078	1.191	1.171	0.084	1.30
RIPK2	1.034	1.110	1.381	1.175	0.182	1.30
G6PC2	1.171	1.146	1.197	1.171	0.026	1.31
PPFIA4	1.148	1.237	1.127	1.171	0.058	1.31
CDC42BPG	1.041	1.107	1.386	1.178	0.183	1.33
MAST3	1.413	1.251	0.837	1.167	0.297	1.33
MVK	1.217	1.145	1.166	1.176	0.037	1.34
FGR	1.179	1.238	1.107	1.175	0.066	1.35
CTDP1	1.222	1.104	1.211	1.179	0.065	1.36
PPP1R15A (§)	1.417	1.290	1.062	1.256	0.180	1.36
PIP5K3	1.151	1.067	1.328	1.182	0.133	1.37
PPP1R13B	1.210	1.224	1.098	1.177	0.069	1.37
PIK3R1	1.097	1.160	1.294	1.184	0.101	1.38
RPS6KB1	1.198	1.161	1.184	1.181	0.019	1.38
PPP1R16B	1.148	1.136	1.285	1.190	0.083	1.43
MAPK1 (§)	1.068	1.667	1.075	1.270	0.344	1.43

LYN	1.121	1.260	1.183	1.188	0.070	1.44
PFKFB1	1.129	1.298	1.133	1.187	0.096	1.44
PAK2	1.064	1.289	1.219	1.191	0.115	1.45
RAF1	1.150	1.214	1.210	1.191	0.036	1.46
ITPA	1.220	1.096	1.267	1.194	0.088	1.47
CDC2L6	1.134	1.088	1.373	1.198	0.153	1.49
ENPP2	1.304	1.191	1.087	1.194	0.109	1.50
SIRPD	1.196	1.278	1.109	1.194	0.085	1.50
MAP2K6	1.186	1.536	0.857	1.193	0.340	1.53
NME1	1.290	1.197	1.112	1.200	0.089	1.54
PSPH	1.139	1.312	1.149	1.200	0.097	1.54
PTP4A1	1.210	1.237	1.155	1.201	0.042	1.54
PPP1R2P9	1.218	1.223	1.165	1.202	0.032	1.55
DUSP7	1.047	1.191	1.392	1.210	0.173	1.57
DTYMK	1.218	1.110	1.298	1.209	0.094	1.58
TSKS	1.112	1.135	1.391	1.213	0.155	1.60
CAMK2B	1.165	1.267	1.201	1.211	0.052	1.61
RYK	1.145	1.417	1.065	1.209	0.185	1.62
ALPK2	1.322	1.081	1.238	1.214	0.122	1.63
FER	1.303	1.166	1.170	1.213	0.078	1.63
MYLK2	1.142	1.182	1.323	1.216	0.095	1.63
MAPKAP1	1.056	1.277	1.319	1.217	0.141	1.64
MAP3K14	1.232	1.343	1.061	1.212	0.142	1.65
CALM1	1.261	1.212	1.180	1.218	0.041	1.67
PIK3R2	1.153	1.162	1.349	1.221	0.111	1.67
NRK (§)	1.361	1.194	1.375	1.310	0.101	1.67
FBP2	1.287	1.224	1.160	1.224	0.064	1.72
SPHK1	1.162	1.448	1.058	1.223	0.202	1.73
BTK	1.175	1.268	1.258	1.234	0.051	1.78
PIK3CB	1.261	1.272	1.160	1.231	0.062	1.78
IRAK2	1.317	1.131	1.270	1.239	0.097	1.82
DAPK3	1.289	1.183	1.247	1.240	0.053	1.83
KSR	1.164	1.403	1.146	1.238	0.143	1.83
PIK3CD	1.123	1.235	1.371	1.243	0.124	1.83
PKN2 (§)	1.068	1.974	1.000	1.347	0.544	1.83
LYK5	1.308	1.537	0.853	1.233	0.348	1.84
INPP1	1.206	1.304	1.223	1.244	0.052	1.87
PNKP	1.292	1.320	1.134	1.249	0.100	1.92
PPEF1	1.301	1.201	1.251	1.251	0.050	1.92
PRKCDBP	1.278	1.281	1.195	1.251	0.049	1.93
UCKL1	1.256	1.270	1.253	1.260	0.009	1.98
DUT	1.235	1.300	1.256	1.264	0.033	2.02

FGFR2	1.343	1.226	1.225	1.265	0.068	2.03
CDKN1B	1.131	1.425	1.244	1.267	0.148	2.04
ABL2	1.270	1.268	1.271	1.270	0.002	2.06
CSNK1A1	1.248	1.294	1.265	1.269	0.023	2.06
LATS2	1.457	1.459	0.907	1.274	0.318	2.16
PTPMT1	1.263	1.203	1.389	1.285	0.095	2.16
EPHA3	1.415	1.193	1.241	1.283	0.117	2.17
PTK2	1.327	1.573	0.951	1.284	0.313	2.23
STYXL1	1.270	1.320	1.299	1.296	0.025	2.26
AK3	1.153	1.194	1.583	1.310	0.237	2.32
SBK1	1.441	1.218	1.262	1.307	0.118	2.35
FGFR4	1.325	1.384	1.210	1.306	0.088	2.36
PTPRJ	1.389	1.291	1.242	1.307	0.075	2.36
DUSP18	1.275	1.421	1.234	1.310	0.098	2.38
ERBB3	1.538	1.232	1.173	1.314	0.196	2.43
CDC2L5	1.220	1.606	1.146	1.324	0.247	2.51
PFKFB3	1.503	1.266	1.213	1.327	0.154	2.52
DUSP15	1.229	1.414	1.398	1.347	0.103	2.65
FLT1	1.283	1.433	1.328	1.348	0.077	2.66
MTM1	1.524	1.149	1.379	1.351	0.189	2.67
CKS1B	1.307	1.458	1.292	1.352	0.092	2.70
CKM (§)	1.139	2.419	1.250	1.603	0.709	2.71
PKLR	1.374	1.172	1.573	1.373	0.201	2.82
EPHB6	1.366	1.414	1.333	1.371	0.041	2.84
CSNK1G3	1.489	1.303	1.324	1.372	0.102	2.85
DUSP8	1.383	1.265	1.487	1.378	0.111	2.87
ACPT	1.335	1.511	1.389	1.412	0.090	3.15
BUB1B	1.392	1.273	1.590	1.418	0.160	3.17
SRC	1.428	1.740	1.148	1.439	0.296	3.40
FGFR3	1.746	1.490	1.156	1.464	0.296	3.60
DUSP23	1.387	1.562	1.519	1.489	0.091	3.74
DGKB	1.599	1.441	1.482	1.507	0.082	3.88
DGKQ	1.550	1.591	1.838	1.660	0.156	5.01
PPP1R15B	1.429	1.822	1.722	1.658	0.204	5.02
CDKN2A	1.403	1.479	2.187	1.690	0.432	5.19
ERBB4	1.872	1.634	1.579	1.695	0.156	5.33
BMPR1B	1.733	1.697	1.813	1.748	0.059	5.70
CDK6	3.886	0.776	1.101	1.921	1.709	7.16
EPHB2	2.170	2.488	2.295	2.318	0.160	10.08
CDK5	4.381	1.206	1.374	2.320	1.787	10.24

Supplementary table S2: Validated screening results of the secondary screen. Normalized ratios of *firefly* (IRES) / *firefly* (cap) luciferase activities in Huh7.5 cells are shown. (Mean ± sd from one experiment with three independent replicates on parallel plates). Screening hits (IRES < 100%; p<0.1, t-test) are highlighted in bold and green.

	% IRES translation		% CAP translation		Ratio IRES/CAP	T-test
Gene	m	sd	m	sd		p
**ROR1**	85.8	21.7	172.5	29.8	0.50	0.015
PCTK3	52.1	16.4	98.8	46.7	0.53	0.177
**NTRK1**	55.0	5.0	97.7	17.8	0.56	0.016
**EPHA5**	53.2	8.6	83.2	4.0	0.64	0.005
PDK4	133.6	27.6	208.7	39.4	0.64	0.054
**MKNK1**	86.3	11.6	126.3	16.2	0.68	0.025
MTMR12	66.5	6.0	91.0	22.2	0.73	0.139
STK25	104.2	41.7	141.4	14.4	0.74	0.217
**PIK4CB**	81.5	13.7	110.0	11.8	0.74	0.052
**NYD-SP25**	82.7	20.8	111.5	5.6	0.74	0.083
**PTPN2**	75.0	8.0	100.9	16.5	0.74	0.071
CDK4	79.1	8.5	100.6	18.4	0.79	0.141
**ULK1**	44.0	3.8	52.3	5.1	0.84	0.087
MGC75495	51.5	17.8	61.2	7.8	0.84	0.438
GK2	109.6	20.0	129.4	57.5	0.85	0.603
CDC42BPA	84.2	8.7	98.6	11.4	0.85	0.157
GRK4	126.8	25.6	146.8	27.0	0.86	0.406
PFKM	76.2	6.7	86.6	21.5	0.88	0.467
PTPN12	65.7	21.9	73.5	24.9	0.89	0.705
KHK	50.6	7.8	56.2	12.5	0.90	0.539
ALPI	82.4	13.2	90.0	25.3	0.92	0.666
ADK	50.7	8.8	55.2	2.2	0.92	0.438
GALK2	120.2	12.5	127.4	44.8	0.94	0.803
CIB3	64.0	19.7	66.9	17.8	0.96	0.857
DGKZ	97.4	17.1	101.7	28.0	0.96	0.835
PTPLA	200.3	36.9	208.5	70.1	0.96	0.866
MST1R	99.7	5.8	103.3	46.5	0.97	0.901
MAP3K4	127.7	3.6	129.4	62.6	0.99	0.966
PTPRN	130.8	20.4	128.5	67.0	1.02	0.956
MAP3K12	91.7	38.2	89.3	11.5	1.03	0.919
PIM1	125.2	10.3	121.5	24.6	1.03	0.825
CDKN2B	181.1	23.1	167.7	35.8	1.08	0.613

LTK	204.4	31.2	188.0	90.3	1.09	0.781
ITPKA	110.2	21.3	97.7	7.1	1.13	0.385
TYROBP	113.1	20.1	97.1	22.6	1.16	0.411
PRKAA2	133.3	10.2	113.8	31.4	1.17	0.364
PFKFB4	149.7	15.6	126.8	38.5	1.18	0.393
PPP1R12C	132.2	30.0	107.7	48.5	1.23	0.499
MINPP1	201.4	20.8	163.1	67.9	1.23	0.403
CSK	158.7	23.7	127.5	16.0	1.25	0.131
CDK7	172.8	26.8	135.6	37.4	1.27	0.234
CDK8	151.3	32.4	114.4	22.4	1.32	0.180
CSNK2B	126.8	17.6	95.4	27.6	1.33	0.173
CKMT2	101.6	18.4	72.6	23.5	1.40	0.168
PTPRS	0.4	0.1	0.2	0.0	2.00	0.007
EPHB2	206.5	27.7	86.6	11.4	2.38	0.002

Supplementary table S3: Impact of gene silencing of screening hits on HCV infection. Impact of gene silencing (four individual siRNAs per target) on HCVcc infection (strain Luc-Jc1) of Huh7.5.1 cells. Data are expressed as mean relative light units +/- sd (one experiment with three independent replicates on parallel plates). Risk-free siRNA (CTRL), siRNAs targeting CD81 or luciferase served as controls and are highlighted in bold. ULK1 was screened in an independent experiment and provided separately together with the corresponding controls.

Experiment 1	Luciferase activity							
Gene	m_si1	sd_si1	m_si2	sd_si2	m_si3	sd_si3	m_si4	sd_si4
**CTRL**	712957	81338						
**CD81**	157138	37465						
**Luciferase**	149347	43331						
CDK4	346713	133756	1347077	353268	925320	247770	365680	101871
EphA5	567350	21988	81030	58168	554297	63572	53183	18384
MKNK1	944740	151081	582957	113181	393997	81617	363567	100588
MTMR12	134013	34771	609040	115988	184533	19418	378613	25276
NEK7	575500	63490	894437	197798	251450	112059	1049873	89200
NTRK1	834343	134365	537073	47967	955840	14587	478640	56582
NYD-SP25	562000	261001	411207	88323	611927	229800	296070	113363
PIK4CB	789647	92954	384170	37430	394263	108481	374160	101302
PTPN2	1041720	29609	573240	65793	452960	85441	554610	23087
ROR1	824177	175535	768230	74890	1006970	200717	699680	83473

CDK4	346713	133756	1347077	353268	925320	247770	365680	101871
Experiment 2	Luciferase activity							
Gene	m_si1	sd_si1	m_si2	sd_si2	m_si3	sd_si3	m_si4	sd_si4
**CTRL**	114143	65491						
**CD81**	1617	1432						
ULK1	107120	42004	83337	8206	16597	3132	5480	3396

Supplementary table S4: Impact of gene silencing of screening hits on cell viability. Huh7.5.1 cells were silenced with siRNA (four individual siRNAs per target) and cell viability was assessed using MTT (Experiment 1) and Presto Blue (Experiment 2) assay. Data are expressed as mean light absorption +/- sd (one experiment with three independent replicates on parallel plates). Risk-free siRNA (CTRL), siRNA targeting CD81 and luciferase served as controls and are highlighted in bold. ULK1 was screening in an independent experiment and provided separately together with the corresponding controls.

Experiment 1	Cell viability							
Gene	m_si1	sd_si1	m_si2	sd_si2	m_si3	sd_si3	m_si4	sd_si4
**CTRL**	0.155	0.031						
**CD81**	0.144	0.025						
**Luciferase**	0.155	0.025						
CDK4	0.129	0.018	0.141	0.029	0.170	0.039	0.131	0.022
EphA5	0.108	0.025	0.100	0.017	0.096	0.010	0.079	0.006
MKNK1	0.139	0.037	0.128	0.021	0.126	0.013	0.122	0.015
MTMR12	0.104	0.021	0.131	0.037	0.112	0.009	0.098	0.009
NEK7	0.147	0.032	0.126	0.025	0.130	0.020	0.182	0.047
NTRK1	0.155	0.061	0.142	0.015	0.129	0.026	0.155	0.028
NYD-SP25	0.151	0.049	0.146	0.022	0.169	0.018	0.119	0.019
PIK4CB	0.121	0.019	0.119	0.020	0.136	0.022	0.147	0.020
PTPN2	0.131	0.026	0.150	0.019	0.128	0.017	0.116	0.022
ROR1	0.134	0.015	0.144	0.010	0.134	0.026	0.180	0.033
CDK4	0.129	0.018	0.141	0.029	0.170	0.039	0.131	0.022
Experiment 2	Cell viability							
Gene	m_si1	sd_si1	m_si2	sd_si2	m_si3	sd_si3	m_si4	sd_si4
**CTRL**	0.214	0.010						
**CD81**	0.190	0.011						
ULK1	0.207	0.018	0.205	0.012	0.188	0.007	0.166	0.013

Supplementary table S5: Validated screening results of the tertiary screen. Analysis of the raw data in supplementary tables S3-4. Impact of gene silencing (four individual siRNAs per target) on HCVcc (strain Luc-Jc1) infection of Huh7.5.1 cells in fold change (fc) and SSMDs (one experiment with three independent replicates on parallel plates). Toxic siRNAs are highlighted in red and are defined by a decrease of cell viability >2x plate median of all standard deviations. Screening hits are scored by products of SSMD values from individual siRNAs and highlighted in green if SSMD<=-1.645 ("fairly strong" inhibition).

Gene	fc_si1	fc_si2	fc_si3	fc_si4	SSMD_si1	SSMD_si2	SSMD_si3	SSMD_si4	Score
**PIK4CB**	1.11	-1.86	-1.81	-1.91	2.176	-3.675	-1.807	-4.706	**10.19**
**MKNK1**	1.33	-1.22	-1.81	-1.96	0.930	-0.334	-2.358	-6.657	**-9.01**
**NYD-SP25**	-2.00	-1.35	-2.78	-1.47	-2.878	-0.541	-5.657	-0.496	**-8.53**
PTPN2	1.46	-1.24	-1.57	-1.29	1.944	-1.461	-41.805	-1.037	
EphA5	1.06	-6.58	1.04	10.03	0.272	-3.969	0.150	-3.151	
NTRK1	1.01	-1.53	1.16	-1.72	0.210	-4.259	1.113	-1.338	
ULK1 (§)	-1.07	-1.37	-6.88	20.83	-0.071	-0.369	-1.176	-1.310	
ROR1	1.00	-1.07	1.22	-1.18	0.054	-0.170	0.518	-0.504	

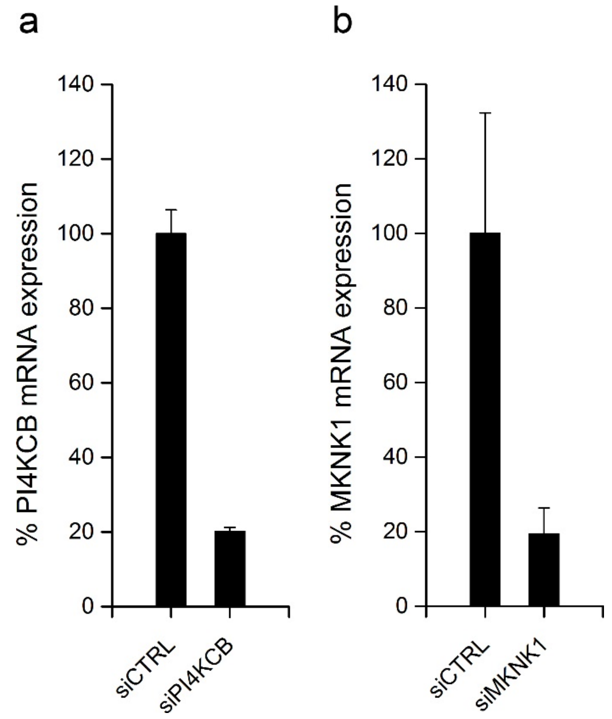

Supplementary figure S1: Specific mRNA knockdown by candidate siRNAs. Quantitative RT-PCR 2 days after gene silencing of PI4KCB (**a**) and MKNK1 (**b**) with specific siRNA (pool of four siRNAs per target). Data are expressed in percent target mRNA expression normalized to GAPDH +/- SEM (one experiment with three independent replicates on parallel plates, measured in duplicates by qPCR).
